# Maternal postnatal bonding and its risk factors: a longitudinal study

**DOI:** 10.1186/s13034-025-00984-4

**Published:** 2025-12-06

**Authors:** E. A. Rusanen, E. M. Vierikko, A. R. Lahikainen, P. L. Pölkki, E. J. Paavonen

**Affiliations:** 1 Faculty of Educational Sciences, University of Helsinki, P. O. Box 9, 00014 Helsinki, Finland; 2 Promotional and Preventive Work Unit, Mental Health Team, Finnish Institute for Health and Welfare, P. O. Box 30, 00271 Helsinki, Finland; 3 Faculty of Social Sciences, Tampere University, 33014 Tampere, Finland; 4 School of Social Sciences and Humanities, Tampere University, 33014 Tampere, Finland; 5 Department of Social Sciences, University of Eastern Finland, P. O. Box 1627, 70211 Kuopio, Finland; 6 Pediatric Research Center, Child Psychiatry, University of Helsinki and Helsinki University Hospital, P.O. Box 400, 00029 HUS, Finland

**Keywords:** Postnatal bonding, Prenatal expectations regarding unborn baby, Stress, Anxiety, Depression, Adverse life events, Family atmosphere, Adult relationships

## Abstract

**Background:**

Mother–infant bonding begins during pregnancy, and a strong bond is crucial for a child's physical and psychological development. The development of this bond is influenced by various factors, including the mother’s psychosocial wellbeing and the support she receives during pregnancy and after childbirth. However, few longitudinal studies have examined this topic, and previous results of cross-sectional studies have been somewhat contradictory. Our objective was to investigate the development of postnatal maternal bonding and to identify the most significant pre- and postnatal psychosocial risk factors that influence maternal bonding at eight months postpartum.

**Methods:**

This longitudinal study examined 1298 mothers. Psychiatric symptoms and social risk factors were assessed using standardized self-report questionnaires. The change in bonding from three months to eight months was analyzed using repeated measures ANOVA. The maternal psychosocial factors associated with bonding at eight months were examined using logistic regression analysis.

**Results:**

We found that maternal bonding improved among all the mothers between three and eight months postpartum. However, mothers who experienced psychiatric symptoms or social problems exhibited weaker bonding at both three and eight months postpartum. The most significant risk factors for bonding issues at eight months were bonding disturbances at three months and a lack of positive expectations regarding the relationship with the unborn baby. Postnatal depression, stress, and anxiety were also linked to an increased risk of bonding disturbances.

**Conclusion:**

We recommend that healthcare professionals focus on identifying difficulties in mother–infant bonding during the prenatal and perinatal periods. They should also consider psychiatric symptoms when assessing the need for support.

## Introduction

The mother–infant bond is important for a baby’s physical, psychological, and social development. Recent studies by Le Bas et al. [1] and Rusanen et al. [2] have reported that both pre- and postnatal bonding are associated with better social emotional development among infants at 12 and 24 months of age. Parental bond is defined as the emotional bond that caregivers develop with their child. According to Condon and Corkindale [3], the core of bonding is the emotional dimension found in the love for one’s child. The parent enjoys closeness with and wants to spend time with their child and get to know their inner world thoroughly. When bonding is strong, the parent protects their child from harm, pain and discomfort and is able to tolerate their child’s various actions, including those that may be irritating [3]. Whereas attachment refers to a child’s bond with their caregiver, bonding refers the caregiver’s (i.e., parent’s) bond with their child.

Maternal emotional availability is a concept related to bonding. According to Bowlby [4, 5], maternal (emotional) availability refers to the caregiver’s physical and psychological presence in the child's life and the mother’s readiness to respond sensitively and reciprocally to her baby’s emotional and interactional signals, so that the child feels secure. In other words, whether the infant feels that the caregiver is emotionally available is shaped through the caregiver’s sensitive responses. Thus, maternal emotional availability refers not only to the emotions that the mother feels (similar to bonding), but also to her sensitive responses toward her child’s signals.

A mother’s bond with her baby is a developmental process that begins before the baby is born [6–8]. It develops further after the baby is born. It has been reported that the mother–infant bond tends to be stable [8]. For example, the prenatal bond and the bond at six and 12 months, and the prenatal bond and the bond at two and 12 months have shown to be connected [7, 9, 10]. Maternal mental health and wellbeing are closely related to the development of bonding. According to a recent meta-analysis, both prenatal and postnatal depressive symptoms are associated with postnatal bonding difficulties [8]. For example, O’Higgins et al. [11] reported that 24.1% of depressed mothers of four-month-old babies have a poor bond with their infant, compared to 5.1% of non-depressed mothers. However, findings regarding prenatal bonding have varied: Prenatal depression was associated with prenatal bonding impairment in only 10 of the 17 studies in a meta-analysis by Tichelman et al. [8]. Although results have been inconsistent, Flykt et al. [12] have concluded that prenatal depression is even more harmful to the quality of dyadic interaction than postpartum depression. More studies are therefore needed to evaluate the role of depression, and they should consider both current and past depressive symptoms.

A few studies have also evaluated whether negative lifetime experiences are related to the development of the mother–infant bond. Fuchs et al. [13] have reported that emotional or physical neglect in early childhood is negatively associated with a mother's emotional availability to her baby. Maternal emotional availability encompasses factors such as sensitivity, structuring, non-intrusiveness, and non-hostility. Specifically, mothers who experienced emotional or physical neglect or maltreatment during their own childhood showed a smaller increase in emotional availability toward their babies between the ages of five and 12 months than other mothers [13]. However, Muzik et al. [14] made contrasting findings, and reported that the maternal bond develops positively during the six months after birth, regardless of the mother’s psychiatric history (e.g., maltreatment, PTSD, and depression). More studies are thus needed to evaluate the role of negative lifetime experiences in the development of maternal bonding.

A lack of pre- and postnatal social support [15] and postnatal partner support [16] have also been associated with difficulties in mother–infant bonding. For example, a lack of social support during pregnancy has been linked to weaker prenatal and postnatal bonding [15]. Maternal anxiety and stress may similarly be related to the development of maternal bonding, but no previous studies have examined their role in the development of the mother–infant bond.

From a clinical point of view, it is important to identify mothers at risk of bonding disturbances as early as possible. However, the factors that modify the risks of depression, anxiety, stress, and bonding problems have not been clearly characterized, nor have the ways in which various interpersonal factors affect the development of bonding. During periods of transition, such as childbirth, various unresolved issues can resurface in a mother’s mind [17], and this can potentially heighten the risk of bonding difficulties. Consequently, pregnancy often presents an opportune moment for therapeutic intervention. Research has indicated that this period can facilitate meaningful therapeutic exchanges, as many parents are motivated to become “good parents” (e.g., [18]). Therefore, there is a pressing need to learn how to identify individuals who would benefit from antenatal parent–infant interaction therapy.

In this longitudinal study, we used multivariate models to investigate how maternal bonding develops when the infant is aged between three and eight months, and how various risk factors are related to changes in maternal bonding. We also computed multivariate logistic regression models to characterize the most significant prenatal and postnatal risk factors for maternal bonding difficulties when the infant is eight months old. The risk factors we included in the statistical models were the mother’s prenatal expectations regarding her unborn baby, adverse life events, depressive symptoms, anxiety, stress, and social risk factors (i.e., poor adult relationships and negative family atmosphere). The study was based on a large population-based sample, and used longitudinal data from pregnancy until the child reached the age of eight months. This study adds to the existing literature by evaluating an extensive set of risk factors for bonding and by characterizing the most significant prenatal and postnatal risk factors that may affect the development of mother–infant bonding.

## Materials and methods

### Sample

This study was based on the CHILD-SLEEP cohort, which represents a general population sample of children’s health, development, and sleep quality [19]. Maternity clinics in central Finland (n = 63) recruited the families during routine visits to maternity clinics at around the 32nd week of pregnancy, and these families were followed for three, eight, 18, and 24 months, and five years after the child’s birth. The participants were asked to give their consent to the study and were informed that they could withdraw at any time. The study protocol was accepted by the local ethics committee (R11032/9.3.2011). Permission for the recruitment procedure was also received from the chief physicians at the targeted health centers.

This study had three time points: prenatal, three months, and eight months. The prenatal data comprised 1667 women, of whom 1421 (85.2%) returned both the prenatal and the three-month postnatal questionnaires. A total of 1298 (77.9%) returned all three: prenatal, three-, and eight-month postnatal questionnaires (Table 1).

**Table 1:** Description of sample

	N	%
Age of mother (years)		
17–25	161	11.5
26–35	1048	74.7
36–48	194	13.8
Disposable income of mother (euros)		
Less than 2000	1025	74.0
2000–3000	303	21.9
More than 3000	57	4.1
Number of previous children		
None (unborn is first child)	556	45.8
One or two	617	50.9
Three or more	40	3.3
Education		
University (highest level)	482	34.1
University of applied sciences (upper secondary level)	533	37.7
Secondary level (lower secondary level)	285	20.1
Vocational qualification(s)	17	1.2
No vocational qualification	68	4.8
Other	30	2.1
Educational status -Basic and vocational education together, i.e., total length of education		
1. About 11 years: comprehensive school + lower vocational education (maximum)	219	16.0
2. 12–14 years: comprehensive school + higher vocational education or + a degree from a university of applied sciences, or a high school diploma + lower vocational education (maximum)	240	17.6
3. 15–16 years: high school diploma + higher vocational education or + a degree from a university of applied sciences	431	31.5
4. About 17 years: comprehensive school diploma or high school diploma + university degree (master’s degree or higher)	477	34.9

### Questionnaires

The questionnaire comprised scales to assess the mother’s expectations regarding her unborn baby, mother–infant bonding, psychological factors (stress, depression, anxiety, and adverse life events), social factors (family atmosphere and mother’s relations with her partner and other adults), health factors, and demographic factors (age, educational status, income, parity). The same scales for psychological factors and family atmosphere were presented at three and eight months postpartum, and those for the mother’s relations with her partner and other adults were presented prenatally. The three- and eight-month questionnaires also contained questions on maternal bonding with the baby.

#### Prenatal measurements

We measured the mothers’ prenatal expectations regarding their unborn babies using a 12-item self-report—the *Representations of Unborn Baby measure (RUB-M*) with a Likert scale [20]. We used these measures of prenatal expectations as indicators of prenatal bonding. We asked the mothers: “What kind of expectations do you have regarding your future baby?” The statements in our questionnaire, such as “I imagine that my baby will be satisfied and happy”, were worded simply to ensure they were easy to understand. The response options ranged from 1 to 5 (1 = not at all…5 = very much).

We submitted the twelve items to maximum likelihood factor analysis and extracted three factors: *Positive expectations regarding the relationship with the baby, Negative expectations regarding caring for the baby*, and *Positive expectations regarding the baby’s eating and sleeping regularity,* as reported previously [21]. These three factor scores were dichotomized at either the 10th or 90th percentile to form the risk and comparison groups [22]. The factor representing positive expectations regarding the relationship with the baby was dichotomized at − 1.39 (risk group ≤ − 1.39; comparison group > − 1.39). The factor representing negative expectations regarding caring for the baby was dichotomized at 1.18 (risk group ≥ 1.18, comparison group < 1.18). The factor representing positive expectations regarding the baby’s eating and sleeping regularity was dichotomized at − 0.93 (risk group ≤ − 0.93, comparison group > − 0.93).

A lack of expectations regarding the unborn baby was also based on the *RUB-M* 12-item self-report. In this measure, responses of “I cannot say” were assigned a code of 1, as reported previously [20]. A summary score was calculated, and its reliability was α = 0.73. The higher the score, the fewer expectations the mother had regarding her unborn baby. Using a cut-off score of ≥ 5, we obtained two groups: mothers with more expectations regarding their unborn baby (comparison group ≥ 5), and mothers with no expectations (risk group < 5) regarding their baby [20].

The mother’s adult relationships (including with their spouses) were assessed using the 18-item self-reported *Adult Attachment Scale* [AAS] questionnaire, which has a 1–5 scale [23]. We used three AAS subscales: (1) *closeness*, which contained five items: for example, “I find it relatively easy to get close to others”, (2) *confidence,* which contained six items: for example, “I know that others will be there when I need them”, and (3) *anxiety*, which contained five items: for example, “I often worry that my partner does not really love me” (scale 1–5, 1 = completely inappropriate, 5 = completely appropriate). Summary scores were calculated. The reliability of summary closeness was α = 0.70, summary confidence α = 0.86, and summary anxiety α = 0.75. We dichotomized the sum scores of these subscales at the 10th or 90th percentile to form risk and comparison groups [22]. The mothers with low closeness scores (≤ 3.20), low confidence scores (≤ 2.67) or high anxiety scores (≥ 2.80) were placed into the risk groups. The mothers with high closeness scores (> 3.20), high confidence scores (> 2.67) or low anxiety scores (< 2.80) were placed into the comparison group [22].

Adverse life events were measured using the *List of Threatening Experiences (LTE),* a scale that consists of 11 items that measure distressing life events [24]. The items were recoded as 1 if they were distressing and 0 if not. The summary score of the items was a cumulative number of distressing events during the past six months and its reliability was α = 0.73. Two groups were formed on the basis of a cut-off score of ≥ 2.

#### Postnatal measurements

*Brockington’s Postpartum Bonding Questionnaire (PBQ)* is widely used to measure mother–infant bonding and has good psychometric properties [25, 26]. In this study, we used one of its four subscales, the general factor, which represents a weakened mother–infant bond and negative attitudes toward the baby [25, 27]. It comprises 12 items and has shown to be reliable and valid [26, 28, 29]. An example item is “I have a close relationship with my baby” (reverse coded), “The baby doesn't feel like mine”, “The baby is getting on my nerves”, “My baby is annoying me”, “I’m unable to accept my baby”. The response options range from 1 to 6 (1 = never…6 = always). Based on these items, the questionnaire measures deficiencies in parent–infant closeness and weak emotional connections with the baby in general, and the inability to accept and tolerate the baby itself and its demands (see 3). We recoded the items as 0–5, in accordance with Brockington et al. [25] and calculated the summary scores. The reliability of the summary score was α = 0.76 at three months and α = 0.78 at eight months. The mothers were classified into two groups using a cut-off score of > 11 to indicate an increased risk of bonding disturbance as defined by Brockington. The mothers at a low risk of bonding disturbance were placed in the comparison group (≤ 11) and the mothers at a high or elevated risk of bonding disturbance (> 11) were placed in the risk group.

We measured postnatal depression using a 10-item version of *the Center for Epidemiological Studies Depression Scale* at both three and eight months postpartum (CES-D; [30, 31]). The response options ranged from 0 to 3 (0 = rarely or not at all or less than once a week, 3 = all the time or 5–7 days per week). The summary scores were calculated at three and eight months. The reliability was α = 0.81 at three months and α = 0.82 at eight months. The scores were then dichotomized using a cut-off score of ≥ 10 (90th percentile) to identify those who had the highest levels of depression.

At both three and eight months, we measured postnatal anxiety using a shortened version of the *Spielberger’s State-Trait Anxiety Scale*, which had seven items, such as, “I am nervous and restless”, “I worry too much about things that don't matter”, “I have disturbing thoughts”, “I get stressed and upset when I think about my current life situation” [32]. The response options ranged from 1 to 4 (1 = almost never…4 = almost always) and the summary scores were calculated at three and eight months. Reliability was α = 0.81 at three months and α = 0.83 at eight months. The scores were then dichotomized using a cut-off score of ≥ 12 (90th percentile) to identify those with the highest levels of anxiety.

We assessed postnatal stress at three and eight months postpartum, using a five-item version of the perceived stress scale, *Global Measure of Perceived Stress* [33]. Examples of its items are “How often have you felt confident in your ability to manage your problems?” (reverse coded), “How often have you felt that adversity piles up to the point where you can no longer cope with it?”. The response options ranged from 0 to 4 (0 = not even once, 4 = very often)*.* The summary scores were calculated, and the reliability was α = 0.77 at three months and α = 0.74 at eight months. The mothers were divided into two groups using a cut-off score of ≥ 11 (90th percentile) to identify those with the highest levels of postnatal stress.

We also evaluated postnatal family atmosphere at three and eight months postpartum using *a seven-item bipolar semantic differential scale:* approving (= 1) to disapproving (= 7), safe (= 1) to unsafe (= 7), paralyzing (= 1) to enthusiastic (= 7), and reserved (= 1) to open (= 7). The summary scores were calculated, and the reliability was α = 0.86 at three months and α = 0.88 at eight months. Two groups were created using a cut-off of > 35 (10th percentile). We used measures of postnatal family atmosphere and prenatal maternal relationships with adults (AAS measure, presented earlier) as indicators of social risk factors.

The demographic factors were the mother’s educational status, age, income, and parity in the family. The educational status variable was formed by considering different levels of education and its changes over previous years, and by combining basic education (primary/elementary school, high school), vocational education (lower and upper vocational education), and higher education (university of applied sciences, university), in order to highlight the overall education of the individual, its length and complexity, and thus the family's socioeconomic status.

### Statistical analyses

First, we evaluated the characteristics of the sample using frequency analysis and studied the distributions of the maternal PBQ scores at three and eight months. We used the *t*-test for dependent samples to compare the PBQ scores at three and eight months and the χ^2^ test to evaluate whether missingness at eight months was related to the PBQ scores at three months. Second, we created longitudinal models to study how the risk factors moderated change over time (predicted change in the PBQ scores at three and eight months) using repeated measured ANOVA. In the longitudinal models, PBQ scores at three and eight months were treated as continuous dependent variables. Explanatory variables included maternal psychiatric symptoms (depression, stress, and anxiety) and family atmosphere at three months, as well as prenatal predictors such as adult relationships, adverse life events, and maternal expectations about the infant. Maternal age, education, income, and parity were included as covariates, given their established associations with postnatal bonding [16]. To further identify predictors of bonding disturbances at eight months, we conducted binary logistic regression analyses. Prenatal and postnatal risk factors were examined separately, while statistically controlling for maternal depression at eight months and relevant demographic covariates. Maternal depression was controlled for because previous studies have found it to be associated with mother-infant postnatal bonding [8, 11, 12]. We used one way-ANOVA and Tukey’s post hoc test. We conducted all the statistical analyses using IBM SPSS Statistics 25.

## Results

The mean age of the mothers was 30.68 (SD = 4.50, range 18–48 years, n = 1403), which corresponds to the age of pregnant women in general in Finland [34]. The participating mothers were more highly educated than women in general in Finland [35]: 34.1% had a university degree and 37.7% had a degree from a university of applied sciences. Only 4.8% of the mothers had no vocational education. About half of the infants (45.8%) were first-borns. Table 1 provides further information on the socio-demographic factors.

The mean of the PBQ scores decreased from 4.84 (SD = 3.68) at three months to 3.89 (SD = 3.49) at eight months (t(1247) = − 11.88, p < 0.001). The prevalence of mothers at an elevated risk of bonding disturbance (total PBQ score > 11) declined from 5.3% (n = 66) at three months to 2.9% (n = 36) at eight months postpartum. Of the mothers who had elevated bonding disturbance scores at three months, 22 (33.3%, n = 66) still reported high scores when the baby was eight months old, whereas 44 (67.0%, n = 66) reported fewer bonding problems at eight months than at three months. Only 14 (1.2%, n = 1182) mothers at a low risk of bonding disturbances at three months reported more problems at eight months postpartum (i.e., scored > 11).

There was some attrition at eight months, as 168 of the respondents who had responded to the questionnaire at three months (13.5%, n = 1248) did not respond at eight months. Of these, 161 (13.6%, n = 1182) belonged to the low-risk group at three months (total PBQ score ≤ 11) whereas 7 (10.6%, n = 66) belonged to the high-risk group (p > 0.05). Thus, the lower prevalence of women at a high risk of bonding disturbances at eight months was unrelated to bonding at three months.

**Fig. 1:**
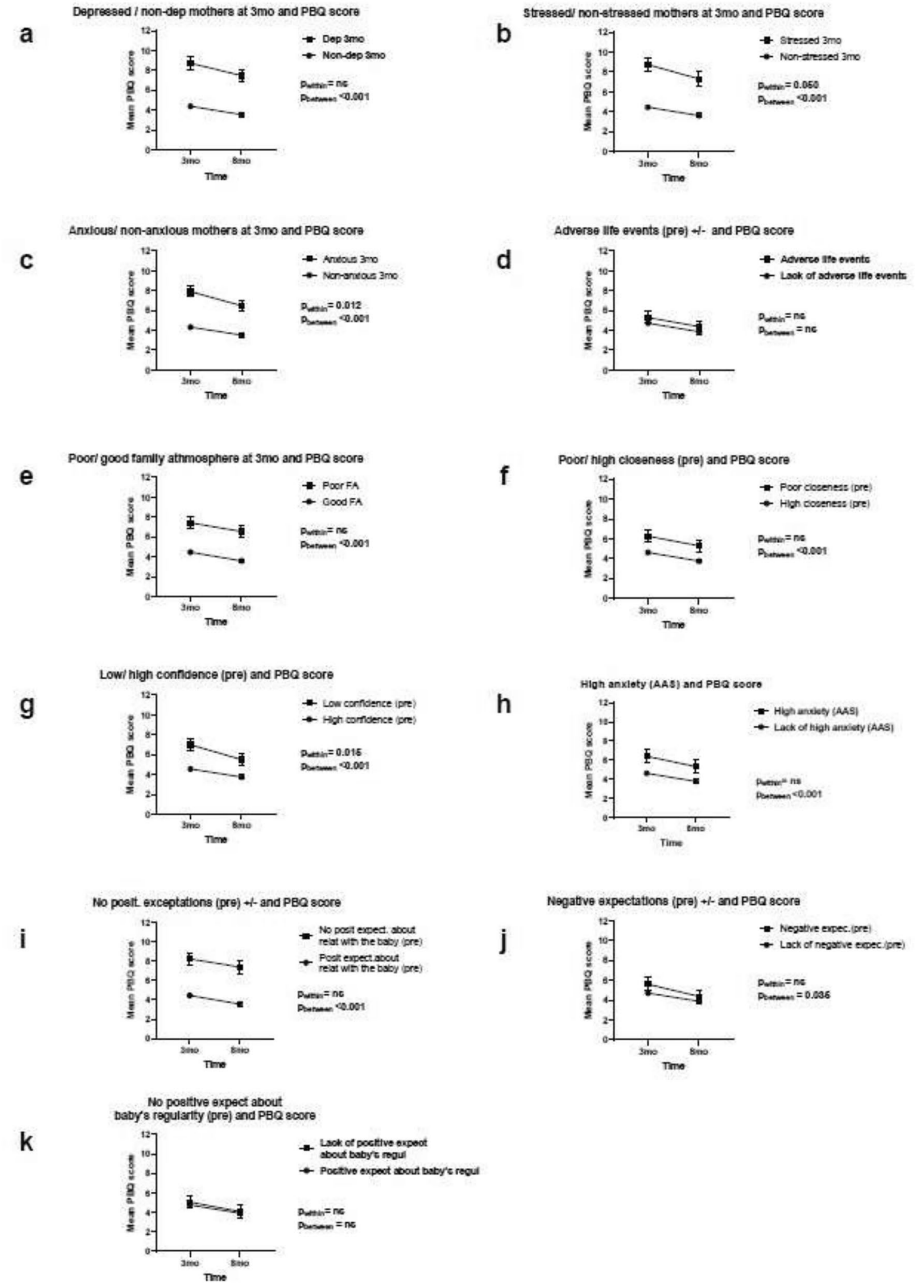
Repeated ANOVA of changes in risk of bonding disturbances (PBQ) over time (3–8 months postpartum). Mother’s age, educational status, parity, and income controlled for

In the repeated measures ANOVA model, we studied whether maternal psychological distress moderated the change in the PBQ scores from three to eight months. The decrease in the PBQ scores was significantly associated with maternal stress (within-subject effect, F(1.000) = 3.84, p = 0.050) and anxiety at three months (within-subject effect, F(1.000) = 6.38, p = 0.012) postpartum, and with poorer prenatal confidence in adult relationships (within-subject effect, F(1.000) = 5.89, p = 0.015). The PBQ scores decreased by a greater amount in the high-risk groups than in the low-risk groups (Fig. 1: b, g, h).

The average PBQ scores (at both time points) were higher among women with depression (between-subject effects, p < 0.001), whose family atmosphere was negative (p < 0.001), who had few close adult relationships (p < 0.001), or whose adult relationships involved a great deal of anxiety (p < 0.001), and among mothers who had few positive expectations regarding their relationship with their baby (p < 0.001) or negative expectations regarding caring for their baby (p < 0.035) (Table 2; Fig. 1). In contrast, adverse life events and expectations regarding the baby’s eating and sleeping regularity during pregnancy were not associated with maternal PBQ scores at three and eight months postpartum (between-subject effects, p > 0.05).

**Table 2:** Repeated ANOVA model of changes in risk of bonding disturbances (PBQ score) from three to eight months. Mother’s age, educational status, parity, and income controlled for

	PBQ scores in low-risk group		PBQ scores in high-risk group		Mean difference	SD	p	Within-subject effect	p	Between-subject effect	p
	Mean 3 months	Mean 8 months	Mean 3 months	Mean 8 months				F		F	
Depression at 3 months*	4.39	3.55	8.62	7.36	− 4.14	0.30	< 0.001	2.05	ns	186.65	< 0.001
Stress at 3 months	4.46	3.63	8.67	7.24	− 3.99	0.33	< 0.001	3.84	0.050	(142.94)	(< 0.001)
Anxiety (State-Trait Anxiety) at 3 months*	4.33	3.53	7.85	6.46	− 3.27	0.26	< 0.001	6.38	0.012	(153.31)	(< 0.001)
Adverse life events, prenatal*	4.74	3.86	5.24	4.32	− 0.55	0.29	ns	0.01	ns	3.50	ns
Negative family atmosphere at 3 months*	4.48	3.59	7.34	6.46	− 2.96	0.29	< 0.001	0.00	ns	101.88	< 0.001
Lack of close relationships, prenatal*	4.63	3.76	6.19	5.22	− 1.59	0.31	< 0.001	0.16	ns	26.19	< 0.001
Low confidence in relationships, prenatal*	4.56	3.75	6.90	5.42	− 2.09	0.32	< 0.001	5.89	0.015	(43.86)	(< 0.001)
High anxiety (AAS), prenatal	4.65	3.80	6.26	5.18	− 1.66	0.34	< 0.001	0.52	ns	23.62	< 0.001
No positive expectations regarding relationship with baby, prenatal*	4.44	3.56	8.31	7.49	− 3.79	0.31	< 0.001	0.02	ns	145.42	< 0.001
Negative expectations regarding taking care of baby, prenatal*	4.71	3.88	5.61	4.38	− 0.68	0.33	0.035	1.91	ns	4.44	0.035
No positive expectations regarding baby’s eating and sleeping regularity, prenatal*	4.78	3.92	4.96	3.99	− 0.22	0.32	ns	0.19	ns	0.49	Ns

Mother’s age (ns), educational status (p < 0.05*), income (ns.), and parity (p < 0.05* controlled for

Next, we evaluated the risk factors for bonding disturbances at eight months and found that the most significant predictive factors were bonding disturbances at three months postpartum (AOR = 21.76, p < 0.001), a lack of positive expectations regarding their relationship with their unborn baby (AOR = 8.58, p < 0.001), as well as anxiety (AOR = 4.01, p < 0.05) and stress at eight months postpartum (AOR = 3.59, p < 0.01), even when we controlled for the background factors and maternal depression at eight months. Adverse life events and social risk factors were not related to bonding disturbances at eight months (Table 3).

**Table 3:** Binary logistic regression analysis models of risk of bonding disturbances (PBQ scores at 8 Months > 11 postpartum). Demographic Factors and Depression at Eight Months Controlled for

Explanatory variables	β	S.E	Wald	AOR (95% Cl)
Risk of bonding disturbances at 3 months	3.08	0.46	44.32	21.76 (8.79–53.88)***
No positive expectations regarding relationship with baby, prenatal	2.15	0.43	24.87	8.58 (3.69–19.97)***
Stress at 8 months	1.28	0.49	6.73	3.59 (1.37–9.43)**
Anxiety (State-Trait Anxiety) at 8 months	1.39	0.54	6.61	4.01 (1.39–11.55)*
Negative family atmosphere at 8 months	0.68	0.46	2.17	1.96 (0.80–4.82)
Adverse life events, prenatal (LTE)	− 0.15	0.59	0.07	0.86 (0.27–2.72)
Lack of close relationships, prenatal	0.20	0.49	0.17	1.23 (0.47–3.20)
Low confidence in relationships, prenatal	0.30	0.48	0.38	1.35 (0.53–3.44)
Anxiety (AAS), prenatal	− 0.24	0.56	0.18	0.79 (0.26–2.36)
Negative expectations regarding taking care of baby, prenatal	− 0.29	0.65	0.20	0.75 (0.21–2.69)
No positive expectations regarding baby’s eating and sleeping regularity, prenatal	− 1.40	1.05	1.79	0.25 (0.03–1.92)
Lack of expectations, prenatal	0.43	0.54	0.63	1.53 (0.54–4.40)

This table presents the explanatory variables one by one, controlled for depression at 8 months postpartum. ***p < 0.001, **p < 0.01, *p < 0.05)

Depression is significant in each analysis (p < 0.01)

Cox & Snell R Square/Nagelkerke R square: Risk of bonding disturbance (PBQ, at 3 months): 0.086/0.369, No positive expectations regarding relationship with baby (at prenatal): 0.07/0.295, Stress (at 8 months): 0.053/0.227, Anxiety (State-Trait Anxiety, at 8 months): 0.053/0.238, Negative family atmosphere (at 8 months): 0.048/0.208, Adverse life events at prenatal (LTE, at 8 months): 0.047/0.201, Lack of close relationships (prenatal): 0.046/0.200, Low confidence (prenatal): 0.047/0.201, Anxiety (AAS, prenatal): 0.046/0.200, Negative expectations regarding taking care of baby (prenatal): 0.047/0.201, No positive expectations regarding baby’s sleeping and eating regularity (prenatal): 0.049/0.211, Lack of expectations (prenatal): 0.047/0.202

## Discussion

In this longitudinal study, we examined the development of maternal bonding from three to eight months and the psychosocial and social risk factors that contribute to the development of bonding disturbances at eight months postpartum. On average, maternal bonding developed favorably during the follow up and the prevalence of bonding problems decreased. Importantly, we noticed that bonding also improved among the mothers with psychosocial risk factors (maternal stress, anxiety, and low confidence in adult relationships). However, the mothers with no psychosocial risk factors reported better overall bonding with their baby (at both time points) than the mothers with psychosocial risk factors. We also found that a lack of positive expectations regarding the relationship with the unborn baby, bonding problems with the baby at three months, and the mother’s stress and anxiety at eight months significantly increased the risk of bonding problems at eight months.

The prevalence of bonding problems was quite low in this sample (5.3% at three months and 2.9% at eight months), which suggests that in most cases, bonding develops well. Only one-third of the women with elevated bonding disturbance scores at three months remained in the high-risk group at eight months postpartum. The low prevalence of bonding problems may reflect how well-developed the perinatal services for parents are in Finland: free maternity clinic services, a well-functioning family policy with child welfare services, and financially secured maternity and family leave [36]. According to Plotka and Busch-Rossnagel [37], the length of maternity leave is associated with the quality of mother–infant interactions, and Clark et al. [38] have reported that a shorter maternity leave is associated with poorer interaction with a newborn baby, and that psychological stressors (such as depression and a baby’s difficult temperament) impair interactions (i.e., sensitivity, responsiveness, and positive affects) between mothers with shorter maternity leave and their babies.

In this study, we evaluated the risk factors for persistent bonding disturbances among mothers. We found that the strongest risk factor for bonding disturbances at eight months was *bonding disturbance at three months,* which increased the risk almost 22-fold. Moreover, maternal reports of *low levels of positive expectations regarding the relationship with the unborn baby* increased the risk for bonding disturbances at eight months postpartum 8.5-fold. This is consistent with previous results, which have suggested that bonding disturbances are often persistent [8]. Our findings add to the previous literature by showing that prenatal expectations are significantly related to mother–infant bonding at eight months, even when demographic factors and depression are controlled for statistically. From a clinical point of view, this finding is particularly important, as it indicates that the mother–infant dyads at risk of bonding disturbances could be screened during pregnancy or when the baby is three months of age.

Also in line with previous studies, we found that postnatal depression is significantly related to bonding problems at three and eight months. The association between maternal depression and perinatal bonding has been reported in several previous studies [8, 14, 16, 39–42]. Depressed mothers may feel unprepared to take care of and detached from their newborn baby, which can affect their ability to interact with it [43]. However, not only depression, but also stress and anxiety were related to more bonding problems at three and eight months. Thus, postpartum interventions directed at mothers and their babies should pay attention to mothers with stress and anxiety in addition to depression. Strengthening maternal bonding can also attenuate the mother’s psychological problems (see [15]).

In addition to psychosocial risk factors, we analyzed how certain social risk factors (family atmosphere and the mother’s relationships with her spouse and other adults) were related to mother–infant bonding problems at eight months. In our study, social risk factors referred to a perceived lack of psychological and emotional support from adults outside and within the family. Neither postnatal family atmosphere nor the mother’s adult relationships were associated with postnatal bonding at eight months postpartum. Bicking-Kinsey et al. [16], for example, reported a positive association between the mother–infant bond and spousal and social support after the baby is born. Ohara et al. [15], in turn, found that a lack of social support can create problems in mother–infant bonding both before and after the baby is born. The reason for these results differing from ours may be that different measures were used. Social support of the mother may be in the form of concrete help or psychological support, which may make its significance for mother–infant bonding different. Social support is an important issue for future studies to consider, as its influence on bonding and parental psychological symptoms has not been sufficiently studied to date.

One of the most important findings of the present study was that mothers with psychosocial risk factors also reported better bonding over time, although their bonds with their babies were generally somewhat weaker at three and eight months postpartum than that reported by mothers with no psychosocial risk factors. Muzik et al. [14] similarly found that bonding also improved from birth to six months postpartum among mothers with psychopathology (e.g., PTSD or depression). Also, Theran et al. [44] reported that weak bonds are more susceptible to change than strong bonds. According to our study, all mothers at risk of bonding problems (regardless of whether or not they have psychosocial risk factors) could benefit from supervision.

This study contributes to the existing research in several ways, but some limitations are worth mentioning. Although the data were from a large birth cohort recruited already during pregnancy, the number of mothers at risk of bonding disturbances was relatively low at both three months (n = 66) and eight months (n = 36), suggesting that the sample was rather normative, with few bonding problems.

One limitation of the study is that it was based on parental reports on bonding. It has been shown that maternal self-reports and observational assessments of the quality of mother–infant interaction (such as maternal emotional availability or caregiving sensitivity) may not correlate. Observational methods can help differentiate between actual behavioral bonding issues and the parent’s personal experiences. However, parents’ subjective concerns about bonding reflect their need for support and interventions and therefore, are usually also valuable in, for example, clinical settings, where interviews are based on similar subjective parental reports. Nevertheless, it is important that future studies also include observational methods, such as emotional availability. As observational methods are also prone to bias, both subjective and objective measurements should be considered as complementary methods in future studies.

We examined the relationship between various factors related to mothers, families, and bonding, but we did not include child-related factors in our analyses, such as an infant’s temperament [45], health [46], and feeding [47] and sleeping patterns [48], which may also have an impact on bonding. Incorporating infant-level variables in future research could help clarify these bidirectional influences.

The study has several strengths. The response rate at all the time points remained relatively high and attrition at eight months was not related to previously reported maternal bonding difficulties. Moreover, as the sample was large, we were able to perform multivariate modeling, and a variety of psychosocial and social factors were taken into account. Including the mother's socioeconomic background (educational status, income) in the analyses strengthened our findings, as previous studies have shown that these factors are related to mother–infant bonding (e.g., [16]).

In conclusion, bonding improved for all mothers after the birth of their child, including mothers with psychosocial risk factors. However, various psychosocial risk factors predicted a higher level of bonding problems at both three and eight months postpartum. Specifically, stress, depression, anxiety, a negative family atmosphere, a lack of close relationships, low confidence in relationships, and high anxiety in adult relationships were associated with more maternal bonding problems at both time points. This study also clearly showed that a lack of positive expectations regarding the relationship with the unborn baby and postnatal bonding disturbances at three months were linked to an increased risk of bonding disturbances at eight months, independent of maternal depression. Our study thus confirms previous findings that bonding problems are often related to various psychosocial risk factors and tend to be persistent. Therefore, in clinical practice, it is important to pay attention to mothers’ difficulties bonding with their unborn baby, psychiatric symptoms, and psychosocial situations as early as during prenatal examinations, and to continue follow up and support after the baby is born. Interventions to support mother–infant bonding should especially be considered in the case of additional psychosocial risk factors.

## Data Availability

The data is sensitive because it contains individuals' medical, psychological, and social information. Thus, in accordance with the General Data Protection Regulation (GDPR), the research data will not be shared.
